# Impact of Titanium Mining and Other Anthropogenic Activities on Malaria Positivity Rates and Parasitemia in Five Selected Study Sites in Msambweni Subcounty, Kwale County, Kenya

**DOI:** 10.1155/japr/6967797

**Published:** 2025-02-05

**Authors:** Edward Githinji, Collins Okoyo, Cassian Mwatele, Juma Mwatasa, Benard Chieng, Sylvie Araka, Henry Kanyi, Sammy Njenga, Judy Mwai

**Affiliations:** ^1^Eastern and Southern Africa Center for International Parasite Control, Kenya Medical Research Institute, Nairobi, Kenya; ^2^Department of Epidemiology, Statistics & Informatics, Kenya Medical Research Institute, Nairobi, Kenya; ^3^Nagasaki University Institute of Tropical Medicine, Kenya Medical Research Institute, Nairobi, Kenya; ^4^Centre for Public Health Research, Kenya Medical Research Institute, Nairobi, Kenya

**Keywords:** *Anopheles* mosquito, anthropogenic activities, *P. falciparum*, titanium mining, transmission

## Abstract

Africa was home to 95% of malaria cases and deaths in 2021. The negative impacts of malaria can be aggravated by social–economic–environmental factors, more so agroeconomic practices such as irrigation, mining, and dam construction. The aim of this study was to investigate the impact of water harvesting, sugarcane farming, and mining activities on *Plasmodium falciparum* positivity rates and parasitemia densities in five selected sites in Msambweni Subcounty, Kwale Kenya. A cross-sectional concurrent mixed methods study was used to collect data. Kwale County was selected due to the high malaria endemicity possibly attributable to the suitable vector habitat characterized by the major anthropogenic activities. The study had five different arms of investigation; the first arm was the control (C), second dam (D) site, third sugarcane (S) site, fourth mining (M) site, and fifth dam–sugarcane–mining (DMS) site. Each of the 1025 consenting participants from 208 households provided a single blood sample for determining malaria prevalence and parasitemia using rapid diagnostic kit and microscopy. Overall, the malaria positivity rate was 22.9% by rapid diagnostic testing (RDT) and 20.1% by microscopy. *P. falciparum* observation by RDT was highest in the DMS site with 33.7% followed by S site with 26.8%, D site with 23.3%, and M site with 17.6%, and the least was the C site with 11.0%. The overall parasitemia density (parasite counts per 200 white blood cells) was 8.4 with a site-specific density of 18.7, 8.6, 7.1, 3.7, and 3.1 for DMS, S, D, M, and C sites, respectively. Univariable analysis of factors associated with malaria infection showed that participants in the DMS site were four times more likely to be infected with malaria (odds ratio (OR) = 4.1, *p* < 0.001) compared to those in the C site. Malaria vector and human host interactions are often enhanced by suitable environmental conditions especially ambient temperature which accelerate parasite growth in the mosquito and humidity. Anthropogenic activities may open up new breeding sites for the vector or increase human–*Anopheles* infective contact hours, hence the different positivity rates and intensities in *P. falciparum* transmission. The study results showed that prevalence of malaria and parasitemia was highest in areas where all the three anthropogenic activities were taking place. In the single-activity site, sugarcane farming predisposed participants to high malaria burden. Characterized relational interplay between these anthropogenic activities and *P. falciparum* parasitemia will be useful in developing tailored strategies towards optimized malaria control interventions in areas with and without anthropogenic activities.

## 1. Introduction

Malaria remains the foremost cause of morbidity and mortality in the tropics [1]. In 2023, over 50% of the world's population at risk of infection and 95% of the malaria burden were reported in sub-Saharan countries [2, 3]. Increased funding for malaria interventions in the last decade has resulted in reduced malaria parasite transmission rates [4]. However, the benefits achieved with the scaled-up antimalaria interventional measures require comprehensive surveillance and monitoring to ensure continued gains in each and every World Health Organization (WHO) member country [5].

A notable increase in malaria prevalence in the coastal region of Kenya according to Kenya National Malaria Indicator Survey (KNMIS) of 2016 jolted the national and county governments to find out what could be the environmental or social economic drivers attributable to the sudden rise of malaria cases [6]. Mosquito and human factors normally determine rates of new infections with malaria parasites [7, 8]. *Anopheles* breeding is often affected by biotic as well as abiotic factors such as land cover, rainfall, humidity, wind, edaphic, altitude, topography, and temperature which can also be used to forecast the risk of malaria transmission [9, 10]. Significant malaria prevalence may be presumed in zones with higher temperatures and substantial amounts of rainfall [11]. These suitable environmental conditions promote higher fecundity in anopheline species as well as rapid malarial parasite reproduction within the mosquitoes' bodies [10, 12]. Coincidentally, no sudden change in soil and climatic conditions had been reported in the recent past [13, 14]. Highly cultivated areas have increased favourable habitats for most of the primary vectors, which prefer sunlight and nonforested areas [15]. Therefore, clearing bushes, keeping of livestock animals, farming food and cash crops, and development of urban centers may affect malaria transmission levels [16]. Although reduced hygienic conditions in urban areas may advance vector breeding in some instances, say for *Anopheles stephensi*, urbanized centers are likely to have reduced suitable mosquito breeding sites [1, 17, 18]. Nevertheless, positive human behaviors such as prompt and accurate testing for malaria and immediate access to antimalarial drugs if diagnosed positive are vital towards reducing risk [19, 20].

Epidemiology of vector-borne diseases is often influenced by livelihood activities and human movements, which impact positively or negatively on malaria risk in its elimination settings [21, 22]. At the same time, economic and social development is being heavily drained by the costly antimalaria campaigns [22, 23]. In high-endemicity areas, there is limited knowledge on how and to what extent extractive industrial projects are impacting or not, the altered epidemiology, and entomology of malaria [24]. Through a variety of proximal and distal factors, mining and other natural resource development and management projects are considered to influence local disease patterns, including malaria [25]. Particularly, malaria vector *Anopheles gambiae* sensu lato (s.l.) had been reported to exploit favourable habitats created during project-related developments in the modified physical environment [22, 26]. Substandard screening and sanitation of poorly constructed sketchy housing structures expose workers attracted by mining projects to infective mosquitoes' bites [27]. Increased vector breeding and human–vector contact were exacerbated by lack of drainage resulting to periurban water bodies (e.g., swamps) [28]. In contrast, companies have an interest in keeping their workforce healthy and thus productive by actively creating malaria control and prevention opportunities ([24, 29, 30]). In the recent past gold, diamond, lithium, and copper mines in Democratic Republic of the Congo had positively impacted on surrounding local communities, socioeconomically [25, 31].

A proposal to commercially exploit titanium deposits located in the Kwale Region was presented by the Canadian company Tiomin Resources Inc. to the Kenyan Government, where the latter approved after years of negotiations [32]. Kenya joined the league of mineral exporters in February 2014 [32]. Sadly, limited health care and disease prevention measures have been established despite the widespread expansion of legal titanium mining in coastal areas of Kenya [15]. Investigations have demonstrated that mine-related conflict often revolves around six thematic areas, namely, land ownership, “unfair” compensation, inequitable resource distribution, deteriorating communal health status and environmental degradation, mine-induced poverty, and human rights abuses [29]. It is often expected that mine developers will as well finance tangible educational, social and health-related projects and services. Impact of the above-mentioned issues can be minimized through corporate social responsibility (CSR) towards full pacification of age-old diseased and impoverished local communities [32, 33].

Incidentally, malaria prevalence in the coastal region had shot from 4% in 2015 to 7.6% (as percentage of all first outpatient visits) in 2017 [34]. This begged the question: What are the contributing factors towards this sudden spike in higher malaria prevalence in the Kenyan coastal region [35, 36]. Fundamental changes in the ability of *Anopheles* mosquitoes to vector malaria may have been caused by shifts in mosquito feeding and resting behavior connected to insecticide-treated bed net coverage [18, 37]. Changes in land use, housing structures, and/or climate could have shifted microclimatic dynamics associated with *Plasmodium* transmission. Mining regions with disused mining sites contribute a lot to malaria endemicity due to displacement and proliferation of mosquito breeding sites, high human migrations, and lack of adequate antimalaria activities [9, 10]. Specific vector control measures must be introduced to decimate this upcoming source of malaria in Kenya [38]. The study was aimed at determining the malaria prevalence and parasitemia density among residents and migrants in the five different sites of investigation, namely, the control (C) site with no dam, sugarcane, nor mining; the dam (D) site, the sugarcane (S) site, the mining (M) site, and the dam–sugarcane–mining (DMS) site.

## 2. Materials and Methods

### 2.1. Study Design

The study utilized a cross-sectional concurrent mixed methods to collect data. Kwale County was purposively selected due to the high malaria endemicity and the titanium mining explorations activities in the region amidst other anthropogenic activities. Areas with no dam, no sugarcane farming, and no mining excavations in Kwale County formed the control (C) site while areas with dams, sugarcane farming, and mining excavations represented the experimental sites. Houses in areas beyond 3 km away from control and experimental sites under study were excluded.

### 2.2. Study Site

**Figure 1:**
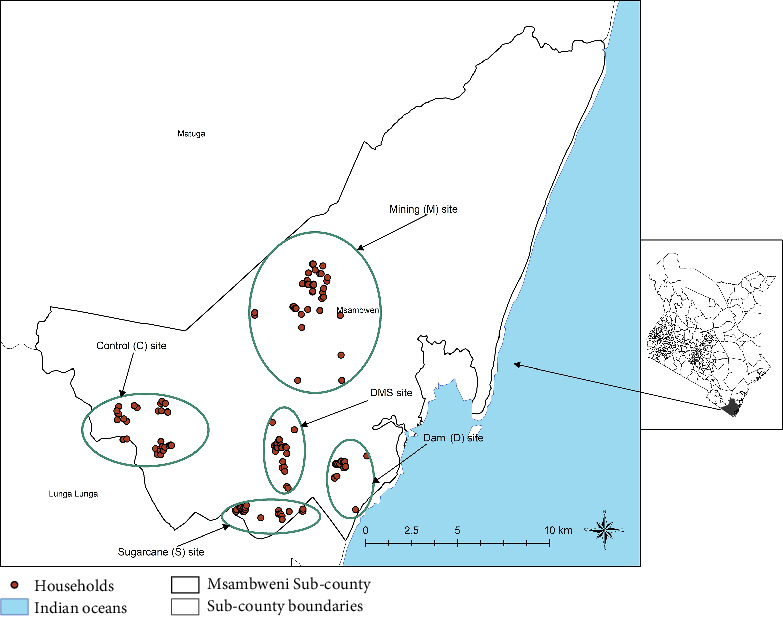
The study site with the four experimental sites and the control site in Msambweni Subcounty, Kwale County, Kenya.

The study was conducted in endemic Kwale County in the coast province of Kenya, a malaria mesohypoendemic zone (Figure 1). Kwale County borders Tanzania to the south-west and the Indian Ocean to the east. The area is often hot and humid all year round with an annual mean temperature range of 22°C–34°C, average relative humidity range of 70%–80%, and annual rainfall range of 900–1500 mm. Altitude ranges from 0 to 462 m above sea level. Both malaria and lymphatic filariasis are endemic in the study area. Kwale residents experience moderate to high malaria transmission. Studies undertaken along the Kenyan coast have reported a yearly increase in malaria admissions and childhood mortalities [13].

### 2.3. Ethical Approval and Consent to Participate

The study protocol received ethical approval from KEMRI's Scientific and Ethics Review Unit SERU No. 3557: “An explorative study on the effect of titanium mining activities on malaria transmission in Kwale County, Coastal Kenya” (version 1.0_dated July 31st, 2017). Additional approval was provided by the county and subcounty-level health authorities after they were appropriately briefed about the study. At household level, individual consent from the household head was sought.

### 2.4. Study Population and Sample Size Determination

Participants of this study were children between 6 months and 18 years, community adult members, and migrant populations. An assent was required for children between 6 months and 18 years while community adult members and migrants were required to give a consent before a sample of blood was drawn from them.

The sample size for the participants was determined using Fischer's formula [39]. Sample size calculation took into consideration the overall malaria prevalence among residents of Kwale County which was 14.1% [40] and a margin error of 5% with 10% nonresponse rates, where *n* was the required minimum sample size, *z* was the score for 5% Type 1 error for a normal distribution (*Z* = 1.96), *p* was the prevalence of malaria in the coast endemic region (found to be 14.1%), *q* = 1 − *p* (85.9%), and *e* was the margin of error set at 5%. The minimum sample size was 186 study participants per village. An additional 10% nonresponse rate was added to raise the sample size to 205 participants per experimental unit or per village.

 $$\begin{array}{c} n=\frac{Z^{2}pq}{e^{2}}=\frac{{\left(1.96\right)}^{2}\times 0.141\times \left(1-0.141\right)}{{\left(0.05\right)}^{2}}=186+\left(10\%\text{of }186\right)=205\text{ children} \end{array}$$

Hence, 205 study subjects were randomly selected per study site per village.

The sampling frame for the study consisted of five arms with a total of 208 households and 1025 participants sampled in the five study villages. Each consenting participant provided a single blood sample for determining malaria prevalence and parasitaemia densities (parasite counts per 200 white blood cells) using rapid diagnostic kit and microscopy. Within each site sampled, 42 houses neighboring the study sites and are within 3-km radius of the sites and over 5-km distance from one site to another were randomly sampled.

### 2.5. Study Populations' Entry, Recruitment Strategy, and Sampling Procedure for the Quantitative Research

Kwale County health management team, its coordinator, and local administration were given a courtesy call, after which village chiefs and elders were consulted and made aware of the study taking place in their areas of jurisdiction. During the meetings with Kwale County health management committee as well as Kwale mining offices, it was found out that Msambweni Subcounty had the highest malaria prevalence in Kwale County and various anthropogenic activities were also ongoing. The C, D, S, M, and combined DMS sites were identified within the Msambweni Subcounty. Sampling in 42 houses was done within 3 km inside the selected sites of economic activities and 5-km buffer zone between any control or experimental study sites after the houses' heads had consented. A total of 1025 study participants were recruited from the 42 houses sampled, among whom were migrant populations from other regions of Kenya and the world, working in the titanium mines. Households were selected using computer-generated random table numbers; study participants were approached and invited to participate in the study. The recruitment was carried out on a rolling basis until the sample size was achieved.

### 2.6. Malaria Sample Collection and Examination

Study participants were asked to consent to give a finger-prick blood sample to the study team. Mothers/caregivers of children between 6 months and 18 years were also asked to consent for their children. A finger-prick blood sample was taken by a trained laboratory technologist and a thick and thin blood slide prepared and labelled with the participant's identification number. The slides were then stained using 10% Giemsa stain and dried. They were then shipped to KEMRI laboratories in Msambweni for microscopic examination. At the laboratory, each slide was examined by two experienced laboratory technologists independently. Further, 10% of the slides were sampled randomly and reread for quality control purposes. Rapid diagnostic testing (RDT) kit *Plasmodium* lactate dehydrogenase and histidine-rich protein detecting *Plasmodium falciparum* (*Pf*(*pLDH/pHRP2*)) was also used for each study subject for the purposes of immediate treatment in case positive for *P. falciparum* as is the ethical practice.

### 2.7. Data Management and Quantitative Data Analysis

Risk factors were analyzed first using univariable analysis allowing for factors associated with malaria infection such as gender and age group and described as odds ratios (ORs), using mixed effects logistic regression at two levels. Throughout the analysis, the participants were nested within houses selected within control and experimental sites. The minimum adequate variables for multivariable analysis were selected using an inclusion criterion of *p* < 0.1. Age was retained as fixed term in the final model regardless of statistical significance due to its known importance. Adjusted odds ratio (aOR) was obtained by mutually adjusting all minimum generated variables using multivariable mixed effects logistic regression at 95% confidence interval (CI), taken into account as hierarchical nature of the data. All analyses were performed using STATA version 14.0 (STATA Corporation, College Station, Texas, United States).

## 3. Results

### 3.1. Study Site Description

The study had five arms, each representing a study site which consisted of one village with an average of 42 households and 205 participants per village. Overall, 1025 respondents in 208 households participated in the study (Table 1).

### 3.2. Malaria Prevalence in the Five Selected Study Sites

**Figure 2:**
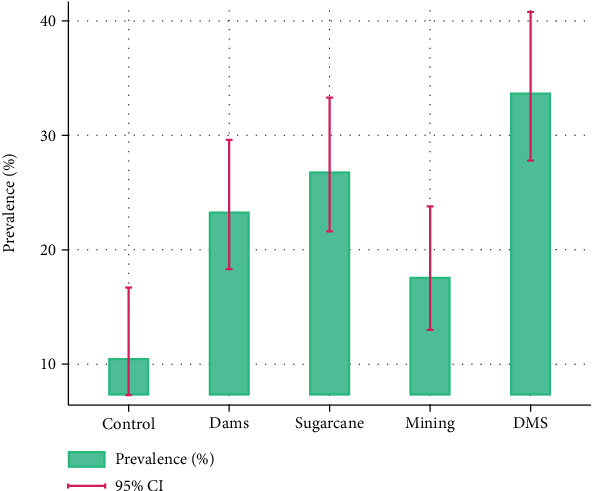
Malaria positivity rate from rapid diagnostic test (RDT) kit results per selected study site in Msambweni Subcounty, Kwale, Kenya.

Overall, the malaria positivity rate was 22.9% (95% CI: 20.4–25.6) by RDT and 20.1% (95% CI: 17.8–22.7) by microscopy. *P. falciparum* observation by RDT was highest in the DMS site 33.7% (95% CI: 27.8–40.8) followed by the S site with 26.8% (95% CI: 21.6–33.4), D site with 23.3% (95% CI: 18.3–29.6), M site 17.6% (95% CI: 13.0–23.8), and the least being the C site with 11.0% (95% CI: 7.3–16.7) (Figure 2).

### 3.3. Parasitemia Density in the Five Study Sites

**Figure 3:**
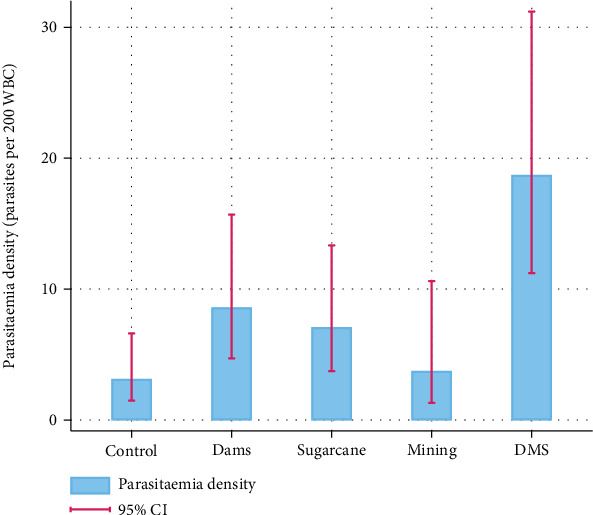
Malaria parasitaemia density from microscopy results per study site in Msambweni Subcounty, Kwale, Kenya.

The overall parasitemia density from microscopy results was 8.4 (95% CI: 6.2–11.3) with a site-specific parasitemia density of 18.7 (95% CI: 11.2–31.2), 8.6 (95% CI: 4.7–15.7), 7.1 (95% CI: 3.7–13.3), 3.7 (95% CI: 1.3–10.6), and 3.1 (95% CI: 1.5–6.6) for DMS, S, D, M, and C sites, respectively (Figure 3). The highest parasitemia density was found in the DMS site where all the selected human activities were undertaken.

### 3.4. Univariable Analysis

Univariable analysis for factors associated with malaria infection showed that overall, participants in the DMS site had significantly higher odds of malaria infection (OR = 4.1 (95% CI: 2.4–7.1), *p* < 0.001) compared to those in the C site (Table 2). The D site and S site also registered significant odds of malaria infection compared to the C site, OR = 2.4 (95% CI: 1.4–4.3), *p* = 0.002, and OR = 3.0 (95% CI: 1.7–5.1), *p* < 0.001, respectively, while the M site had a nonsignificant odds of malaria infection, OR = 1.7 (95% CI: 1.0–3.1), *p* = 0.073.

### 3.5. Multivariable Analysis

The analysis included factors such as the study site and gender (Table 3). The C site and female gender were the reference. In overall, factors significant at univariable model were insignificant at multivariable model as levels of significance were greatly reduced because of the many other factors beyond study site and gender which were put and considered during analysis using the multivariable model. Inhabitants living in the DMS site (OR = 2.1 (95% CI: 0.8–5.5), *p* = 0.144) still had the greatest risk of being infected with malaria parasites followed by the D site (OR = 1.4 (95% CI: 0.5–4.1), *p* = 0.507), though not significant.

### 3.6. Result Summary

Malaria prevalence was highest in areas with all the three anthropogenic activities explored in this study. Moreover, the prevalence of malaria in areas with titanium explorations in Kwale County was relatively low compared to the areas with all the three activities, that is, dams, sugarcane plantations, and mining taking place. Parasitemia density was also low in areas with titanium explorations compared to areas with all the three activities, a good indicator that each of the human activities was a driver towards heightened malaria morbidity and burden.

## 4. Discussion

Dam creation is often aimed at storing water for use domestically in cooking and washing activities and agriculturally in irrigating gardens and farms. Incidentally, such dams become habitation grounds for water living animals and plants. Among the water-living animals are the mosquitoes in the larva stage which mature and later as adult forms transmit various pathogenic organisms [10]. Of our interest is the transmission of *P. falciparum* by *Anopheles* mosquito. Mosquitos are known to oviposit their eggs in fresh or salty water bodies [41].

On the other hand, humans, animals and plants need water to quench their thirst. Green plants are the source of cell sap for male mosquitoes which in turn fertilize female mosquitoes for the latter to oviposit fertilized eggs which hatch into aquatic free-swimming larvae. Mosquito larva growth depends on watered niches with dissolved nutrients [42]. Mosquito larvae are habitual filter feeders. They have fan-like mouth brushes that sweep water at their mouths from in front of them. In this mechanism, they sieve out small particles of decomposing materials and microorganisms, which they then eat. Some larvae in Stages 3 and 4 are known to eat other mosquito larvae in Stages 1 and 2 [43]. Female adult mosquito populations are hematophagous and hence suck blood from humans and animals within 5-km radius or even further if carried away with the help of strong coastal monsoon winds. Humans and animals coming to draw water or drink, respectively, are precious blood-carrying commodities to the malaria vector. The blood meal yields high levels of iron as hemoglobin in erythrocytes and as ferric transferrin [43]. The female mosquito uses the heme-iron part and the globulin-protein part to supply nutrients for viable egg production. Most importantly, iron is required for optimal egg development and viable offspring. Therefore, water environments are crucial for malaria vectors to thrive. Particularly, presence of a dam not only provides mosquitoes with breeding environments but such sites also attract humans and animals for their blood-feeding activities towards self-sustenance and generational continuity [44].

Sugar faming involves land selection, farmer recruitment, land preparation, seed cane stem seed selection and planting, crop maintenance, irrigation in some regions, harvesting, transportation, and cane weighing [16]. Land selection and preparation often necessitate uprooting of trees, clearing of bushes, and plowing and harrowing the land until a clear land is suitable for sugar stem seed planting, activities which engage machines and humans as well. Shallow puddles and pools of water before or during planting of sugarcane are often fertile sites for oviposition of malaria vector eggs, and larval occupation and growth into mature pupa and adults emerge. Subsequent activities equally demand animal, human, or mechanized labor, situations which offer continued blood meal sources for the female mosquitoes. Sugarcane stem and leaves also provide feeding points for male mosquitoes, hence the increased potency of their longer and slender sperms leading to higher fecundity [45, 46]. Both larval nutrition and adult nutrition significantly affect mosquito fecundity [47]. It is known that blood meal quantity and source can influence mosquito fecundity [48]. Intake of carbohydrates can influence egg production while blood meal source and quantity taken by adult mosquitoes determine fecundity [48]. Filled up ventral diverticulum or crop with plant sugar meals can compete for space in the midgut for blood meals and thereby reduce blood meal intake and fecundity among mosquitoes [49]. Sugarcane stems, leaves, and floral parts are also resting places for both male and female mosquitoes. Digesting of blood by female mosquitoes is usually an energy-expending process, hence the nonflight restful mode and posture during these midgut-specific miR-1174 functions of the midgut through its target gene *serine hydroxymethyltransferase* (*SHMT*) enzyme whose depletion causes female mosquitoes to lose their flight ability and die within 48 h of a blood meal [50, 51]. Therefore, rest for female mosquitoes is very important for effective digestion of a blood meal and viable egg production.

Modern mining procedures involve prospecting for ore bodies, analysis of the profit potential of a proposed mine, extraction of the desired materials, and final reclamation or restoration of the land after the mine is closed [22]. During prospecting of crude ores, various technologically identified suspected earth points are opened up, most of which yield negative results leading to movements to fresh grounds and repeating the same procedure until a positive crude ore is discovered. Once discovered, the digging up using earth-moving tools and machines is normally heightened. Micro as well as macro freshly opened up earth points are often left gaping wide which fill up with water once rains fall. The stagnated water shelter, broods, and breeds millions of malaria vector eggs which hatch into larvae [52, 53]. Larvae in turn produce pupae and adults. When female mosquitoes quest for blood meals and suck *Plasmodium*-infected blood meal from humans and animals, with more than a minimum number of the malaria agents enough to cause an infection, the sporogonic cycle of parasites' multiplication in the mosquito begins, while in the mosquito's stomach, the microgametes penetrate the macrogametes generating zygotes. The zygotes in turn become motile and elongated (ookinetes) which invade the midgut wall of the mosquito where they develop into oocysts. The oocysts grow, rupture, and release sporozoites, which make their way to the mosquito's salivary glands. Inoculation of the sporozoites into a new human host perpetuates the exoerythrocytic and erythrocytic stages of malarial agents' life cycle. Workers in mines includes miners, office and security staff, transporters, supervisors, managers, visitors, caterers, and business men and women directly or indirectly related to the mining activities [22]. These workers not only supply blood meals to mosquitos but also provide malarial agents to the blood-questing female mosquito if one is suffering from malaria.

Proceeds from mining are used by the benefitting community to improve infrastructural, social, and educational amenities [54]. Good roads and means of transport contribute to faster access of health centers when one is seeking treatment. Financial enablement allows community members to purchase drugs as well as vector control gadgets such as insecticide-treated nets, repellants, and coils. Stone-walled and concrete-floored houses have been found to reduce indoor mosquito densities. Screened windows, ventilations, and eaves cut off malaria vector access to humans living in the building structure, hence reducing human–mosquito contact hours. Access to primary, secondary, and tertiary education equips community members with knowledge, skills, attitudes, and practices leading to behavior change towards disease diagnosis, treatment, prevention, and control, including malaria. A relationship exists between a positive change in knowledge, attitude, and practices and decline in malaria prevalence [22].

**Figure 4:**
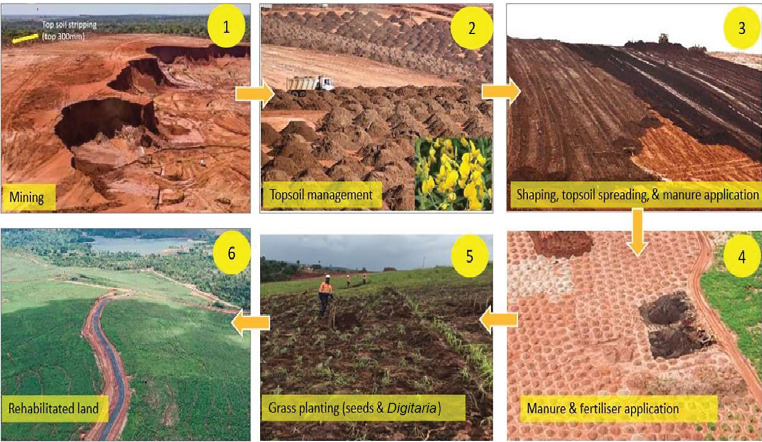
Base Titanium mine dune rehabilitation process (courtesy of Tiomin mining company limited in Msambweni Subcounty, Kwale, Kenya).

**Figure 5:**
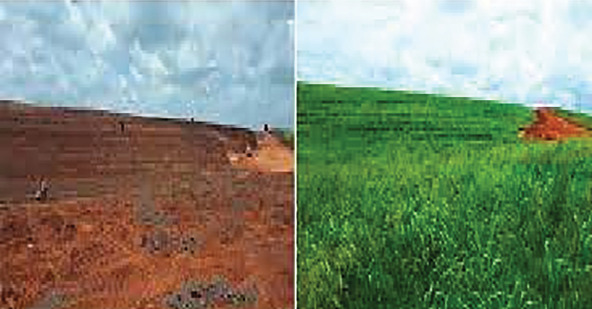
The resultant rehabilitated mine dunes available for agricultural or recreational activities (courtesy of Tiomin mining company limited in Msambweni Subcounty, Kwale, Kenya).

Social corporate responsibility by the mining company includes environment issues such as mined site reclamation and rehabilitation. We found out a well-executed Tiomin mining company reclamation and rehabilitation of mine dune process which were later returned to the community for reuse as agricultural land, recreational activities, and/or man-made forest reserves (Figure 4). Indigenous and exotic grass, herbs, and/or trees were planted and well taken care of resulting into rush green grass lawns and forests which were environmentally fit for habitation (Figure 5).

All this may explain why the study populations in the DMS site had the highest *Plasmodium* positivity and parasite burden. The interplay between the environment, climate, and living organisms triggers a chain of exchanges of malarial agents between living organisms and vectors [5]. Correspondingly, agricultural livelihood activities, travelling, and outdoor social gatherings are linked to increased risk of malaria infection due to prolonged contact with mosquito vectors where standard preventive interventions may not be particularly effective. Notably, movements related to agriculture-like large-scale migration of laborers searching for economic opportunities play role in malaria epidemiology.

### 4.1. Study Limitations

Several limitations were noted in this study design and implementation. First, this is a small case–control study in a confined geographical area with exceptional internal context that provides limited external validity. Second, though the study focused on subjects that were found in their resident areas, hence those who went to work early in the morning and came back in the evening were not captured in this study. Lastly, the use of RDTs might have led to misclassification bias, but per malaria elimination program protocol, all negative RDTs still had a thin blood slide and all positive cases had a second microscopy reading to be evaluated.

## 5. Conclusion

Malaria morbidity and burden were highest in the combined study site of dam, sugarcane, and mining human activities. Sugarcane farming and dam construction were a significant driver in *Plasmodium* sp. prevalence as well as parasite burden, as compared to C and M site; hence, anthropogenic activities affect positivity rates and parasite densities. Therefore, the higher the anthropogenic activities, the higher the malaria incidences and burden. Well thought out, tailored, planned, and implemented mitigation measures per anthropogenic activity can reduce malaria transmission intensities as demonstrated in the titanium study site. Mitigation measures such as flattening excavated sites, rehabilitating mine dunes by planting grass and trees, and prompt malaria diagnosis and treatment by a titanium mining company did bear fruits and resulted to reduced malaria infection rates. Even with continued social economic anthropogenic activities, residents can still live with insignificant *Plasmodium* infections and mosquito bites if proper mosquito control measures are seriously, intentionally, and deliberately undertaken by both the public and private sectors and partnerships thereof.

## 6. Recommendation

Characterized relational interplay between these anthropogenic activities and *P. falciparum* parasitemia may be useful in developing tailored strategies towards optimized malaria control interventions in areas with or without anthropogenic activities. Against new infections, residents may use long-lasting insecticide-treated nets (LLINS), wear body covering clothes, and use insecticide repellants, screened windows and eaves, or closed eaves which have been demonstrated to be still effective as it was in the wealthier mining populations in the M site. To reduce vector densities, residents may use larvicides or biocontrol agents such as *Bacillus thuringiensis israelensis* (*Bti*) in larval breeding sites in sugar farms, surrounding water reservoirs, and flattening and rehabilitation of mining areas after excavations as well as planting grass and trees in reclaimed sand dunes, as done by a titanium (Tiomin) mining company in Msambweni County, Kwale, Kenya.

## Figures and Tables

**Table 1:** The research study site arms and numbers of households and participants in Msambweni Subcounty, Kwale County, Kenya.

**Arms**	**Study site**	**Village**	**Description**	**Number of households**	**Number of participants**
Arm 1	Control (C) site	Mafisini	No mining, no dams, and no sugarcane plantation activities	45	182
Arm 2	Dam (D) site	Marigiza	There were only dam's activities but no mining nor sugarcane activities	42	219
Arm 3	Sugarcane (S) site	Fahamuni	There were only sugarcane plantation activities but no dams nor mining activities	42	220
Arm 4	Mining (M) site	Mwaloya	Only mining activities were in this site. No sugarcane plantation nor dams	43	199
Arm 5	Dam + mining + sugarcane (DMS) site	Gonjora	All the three activities were present	36	205
			Total	208	1025
			Difference between arms (chi-square test, *p* value)	*χ * ^2^ =15.00, *p* = 0.241	*χ * ^2^ = 20.00, *p* = 0.220
				Therefore, different arms were evenly represented at both the household and participant levels	

**Table 2:** Univariable analysis.

**Factor**	**Number examined (number positive)**	**Odds ratio (OR) (95% CI)**	**p** ** value**
*Study site*			
C	181 (20)	Reference	—
D	219 (51)	2.4 (1.4–4.3)	0.002⁣^∗^
S	220 (59)	3.0 (1.7–5.1)	< 0.001⁣^∗^
M	199 (35)	1.7 (1.0–3.1)	0.073
DMS	205 (69)	4.1 (2.4–7.1)	< 0.001⁣^∗^
*Gender*			
Male	428 (115)	1.5 (1.1–2.0)	0.009⁣^∗^
Female	597 (119)	Reference	—

⁣^∗^The statistically significant figures < 0.05.

**Table 3:** Multivariable analysis.

**Factor**	**Adjusted odds ratio (aOR) (95% CI)**	**p** ** value**
*Study site*		
C	Reference	—
D	1.4 (0.5–4.1)	0.507
S	1.1 (0.4–3.3)	0.800
M	1.2 (0.4–3.5)	0.702
DMS	2.1 (0.8–5.5)	0.144
*Gender*		
Male	1.3 (0.7–2.3)	0.335
Female	Reference	—

## Data Availability

The datasets supporting the conclusions of this article are available upon request.
